# The Practical Training of the Territorial Voluntary Aid Detachments

**Published:** 1912-11-30

**Authors:** 


### THE PRACTICAL TRAINING OF THE TERRITORIAL VOLUNTARY AID DETACHMENTS.

In a recent number of The Hospital we outlined the practical test to which the Royal Army Medical Corps had been subjected during the Aldershot Army Manœuvres last summer with the view of ascertaining its efficiency in the two important duties, first, of rendering aid to the wounded on the battlefield, and, secondly, of providing suitable temporary accommodation for them in the shape of a clearing hospital after their removal from the field ambulances. In the medical service of the Territorial Army it will be remembered that the last of these two duties falls to the lot of the voluntary aid detachments. It is they who have to take over the wounded of a division from the Territorial field ambulances, and then to furnish them with proper care and shelter in temporary and clearing hospitals until they can pass them on by train or otherwise to the general hospitals at the base.

### Some County Tests.

To fulfil these obligations satisfactorily implies the need of a practical knowledge of the work. The detachments should be efficient in preparing carts and other vehicles provided by the transport authorities for the removal of lying-down patients and in improvising stretchers. They should be skilled in converting country houses, farms, public buildings, and, in fact, whole villages or small towns into clearing and temporary hospitals. They should be able to utilise local resources in the vicinity of the field of active operations, and to form rest and evacuating or distributing stations, at the former patients receiving rest and refreshment, and at the latter being classified as regards the accommodation they will need on being redistributed, some being sent to one general hospital and others to another. We are glad to see that different counties are taking steps to test their detachments practically in these duties, and we have been impressed by the very complete and thorough demonstration given last July by the Aldershot, Basingstoke, and Hartley-Wintney Detachments of the Hants Branch of the British Red Cross Society. In it there was a combination of the transport, hospital treatment, and rationing of the wounded, which was really the characteristic feature of the display, while care was taken that all the arrangements were carried out as closely as possible to war conditions. Other detachments, when going through their outdoor training next year, might very well follow the procedure adopted, as it furnishes experience in all that is expected from a voluntary aid detachment. In arranging for any such voluntary aid display the following points may be of some assistance in framing and carrying out advantageously the scheme decided on.

When planning a demonstration by voluntary aid detachments in field work there are one or two preliminary points that require attention. The first one is that some days previously a general warning should be issued by the county director to the detachments concerned that there may shortly be hostilities in their neighbourhood, and that they may be called upon to receive a large number of wounded, naming the places where clearing hospitals and rest stations might have to be formed, and reminding them that all available stretchers, hospital equipment, and local transport should be inquired about and earmarked, the transport being possibly needed should the railway traffic be too congested to allow of the conveyance of the wounded by train. The other point of importance is that the day for the combined operations should be one convenient to the detachments, and should be one fixed on after consultation with them. It is the duty of the county director to see that these two details are attended to, as he is responsible for the working of the detachments in his county. He might also make the despatch of the warning notice to the detachments the opportunity for certain general directions, such as the following: (1) That all the medical men will work with their own detachments; (2) that four of the men's detachment must assist the cooks in collecting firewood, making fires, and building shelters, the remainder to form stretcher parties under the doctor and quartermaster; (3) that four of the women's detachment are to act as cooks and be under the quartermaster, the other members to do duty as nurses under the orders of the lady superintendent.

### Necessary Preparations.

Having been warned in this way, the detachments should employ the next few days in getting ready for the special bit of work they will be called upon to carry out. Thus they should arrange for the use of stretchers, splints, bandages, dressings, beds and bedding, furnishings, and, in short, all that may be needed in a clearing hospital. On the evening before the day fixed for the demonstration notice should be sent by the county director to the commandants of the different detachments informing them of the exact emergency that has happened and with which they are to deal. On receipt of this information the commandants will at once issue orders through the quartermasters for the detachment to assemble at a certain time and place, and they will add any further directions that they may consider necessary, as, for instance, any arrangements that have been made for the conveyance of the detachment to the scene of operations, together with general instructions as to the course of procedure on arrival. Should there be more than one detachment it would also be necessary to intimate who is to be in command. It is at this time the work of the quartermasters will be specially onerous, for, after notifying all the members of the time and place of assembly, they have to get together all the necessary stores and equipment and arrange for their despatch to the scene of operations.

On arrival the detachment will at once proceed to work. The men will form stretcher parties for collecting the wounded, while the women will commence at once to equip the temporary hospital decided on, preparing beds, padding splints, getting ready dressings, splints, and instruments, and doing all that is necessary for the reception of the patients. After all the wounded have been brought into hospital, attended to, and fed their subsequent management will depend on circumstances. If they are to be despatched by rail, trucks and vans will have to be specially prepared for them by the men's detachment. This being done, they will be placed in the extemporised ambulance train and sent off, accompanied by a doctor and nurses, when they will be taken to some more permanent hospital and then distributed to the Territorial general hospitals.

### The Hampshire Scheme.

It was such a scheme as this that was carried out by the Hants Branch of the Red Cross Society. A field day was held by some troops from the Aldershot command, and 150 wounded were brought to Fleet. Here they were sheltered in five temporary hospitals formed by the Aldershot and Hartley-Wintney Detachments, and from there conveyed in an extemporised ambulance train of cattle trucks and passenger carriages to Basingstoke, where the train was met by a convoy of carts and two of the local detachments. The wounded were taken from the train, put into the carts, and conveyed either to the Town Hall, which had been converted by two of the local detachments into a hospital of thirty beds, or to the Drill Hall, which had been fitted up by two other detachments as a hospital of seventy beds.

We have only been able to give a brief summary of the operations, but sufficient has been said, we think, to show that the scheme was in every way admirable for furnishing practical instruction, and might well serve as a model to work by. It gives practice in the necessary preliminary preparations needed, in arranging for the assembly and distribution of the detachments, in collecting the wounded, in nursing and feeding them in a temporary hospital, and in their subsequent removal to more permanent sick quarters, while it emphasises the need for the conjoint aid of both the men and women detachments. To carry out all the above duties intelligently and carefully cannot but be a valuable experience, and we hope that all the counties will exercise their detachments on the above lines in the ensuing summer.

